# Knockout of Apolipoprotein E in rabbit promotes premature intervertebral disc degeneration: A new *in vivo* model for therapeutic approaches of spinal disc disorders

**DOI:** 10.1371/journal.pone.0187564

**Published:** 2017-11-03

**Authors:** Anja Beierfuß, Hermann Dietrich, Christian Kremser, Monika Hunjadi, Andreas Ritsch, Thomas Rülicke, Claudius Thomé, Demissew Shenegelegn Mern

**Affiliations:** 1 Central Laboratory Animal Facility, Medical University of Innsbruck, Innsbruck, Austria; 2 Department of Radiology, Medical University of Innsbruck, Innsbruck, Austria; 3 Department of Internal Medicine I, Medical University of Innsbruck, Innsbruck, Austria; 4 Institute of Laboratory Animal Science, University of Veterinary Medicine Vienna, Veterinaerplatz 1, Vienna, Austria; 5 Department of Neurosurgery, Medical University of Innsbruck, Innsbruck, Austria; University of Crete, GREECE

## Abstract

Intervertebral disc (IVD) degeneration that accelerates the loss of disc structural and functional integrities is recognized as one of the major factors of chronic back pain. Cardiovascular risk factors, such as deficits of apolipoproteins that elevate the levels of cholesterol and triglycerides, are considered critical for the progress of atherosclerosis; notably in the abdominal aorta and its lumbar branching arteries that supply lumbar vertebrae and IVDs. Obstruction of the lumbar arteries by atherosclerosis is presumed to promote lumbar disc degeneration and low back pain. APOE-knockout rabbits have recently been shown to generate hyperlipidemia with increased levels of cholesterol and triglycerides that mimic the symptoms of atherosclerosis in humans. Here, we analysed IVD degeneration in the lumbar spines of ten homozygous APOE-knockout and four wild-type New Zealand White rabbits of matching age to prove accelerated IVD degeneration in APOE-knockout rabbits, since APOE-knockout rabbits could be a beneficial model for therapeutic approaches of degenerative IVD disorders. Experiments were performed using T1/T2-weighted magnetic resonance imaging, 3-(4,5-dimethylthiazol-2-yl)-2,5-diphenyltetrazolium bromide assay, glucose-oxidase assay, enzyme-linked immunosorbent assay, quantitative reverse transcription PCR and western blot. APOE-knockout lumbar spines showed more advanced IVD degeneration, obstructed lumbar arteries and lower enhancement of contrast agent in IVDs. Moreover, lower concentration of glucose, lower number of viable cells and lower concentrations of aggrecan, collagen II and higher concentration of collagen I were detected in APOE-knockout IVDs (p < 0.0001). APOE-knockout in rabbits could induce structurally deteriorating premature IVD degeneration that mimics the symptoms of accelerated IVD degeneration in humans. APOE-knockout rabbits could be used as beneficial model, as they can bypass the standard surgical interventions that are commonly applied in research animals for the induction of enhanced IVD degeneration. Their parallel use in therapeutic approaches of IVD disorders and atherosclerosis could reduce the number of research animals to be used and contribute to the principles of 3Rs (Replacement, Reduction and Refinement).

## Introduction

Back pain is a prevalent disorder that affects the majority of adults at some time in their lives [1]. Chronic back pain has been described as part of the global burden of disease that causes immense activity limitations on individuals, which has a huge socio-economic impact in health care and disability compensation [2–3]. Intervertebral disc (IVD) degeneration, which accelerates the loss of disc structural and functional integrities, is recognized as one of the major factors of chronic back pain [4–5]. IVD degeneration is characterized by structural failures that are often accompanied by inflammatory and patho-immunological processes [6–9]. Current treatment modalities are mainly conservative or surgical, and in many cases the therapies are considered insufficient for biological regeneration. In the pathogenesis of IVD degeneration both genetic and environmental factors have been described to play important roles [5, 9–10]. Among other factors, cardiovascular risk factors such as high levels of serum lipids, low levels of apolipoproteins, obesity, diabetes, hypertension and smoking have been considered to promote the progress of lumbar disc degeneration, herniation and radicular pain [11–12]. High levels of cholesterol and triglycerides are considered critical for the development of atherosclerosis; and atherosclerosis of the lumbar arteries is presumed to promote lumbar disc degeneration and low back pain [13–16]. In a long-term follow-up study involving 4886 office-based civil servants (35–55 years of age), who suffered from back pain, low levels of Apolipoprotein A1 (APOA1) and high levels of triglycerides were detected [13]. Moreover, in three distinct long-term follow-up studies involving patients with low back pain high levels of very low density lipoprotein (VLDL) and low density lipoprotein (LDL) were confirmed [14–16].

Apolipoprotein E (APOE) is an important apolipoprotein of chylomicrons (the ultra-low density lipoprotein, ULDL), VLDL, intermediate density lipoprotein (IDL) and LDL. It serves as a ligand for their receptors and is vital for the metabolism of triglyceride-rich lipoproteins (TGRL) as well as for the transport of cholesterol and other lipids in the blood stream [17]. Deficiency of APOE or mutation in APOE that inactivates its binding capacity to lipoprotein receptors can cause type III hyperlipoproteinemia (HLP) [18–21]. Type III HLP causes elevated levels of plasma cholesterol and triglycerides, due to impaired clearance of chylomicrons, VLDL and LDL remnants, which can promote premature development of atherosclerotic plaques [17, 21–23]. Moreover, high levels of cholesterol and triglycerides formatting atherosclerotic plaques have been shown to obstruct the abdominal aorta and its lumbar branching arteries that supply the lumbar vertebrae and IVDs [24–28]. Formation of atherosclerosis starts at an early adult age and its rapid growth in the abdominal aorta occurs between 44 and 64 years of age, which is coinciding with the emerging and advancing processes of IVD degeneration [29–30].

Because of their susceptibility to develop atherosclerosis as well as their similarity in lipid metabolism and cardiovascular physiology to that of humans, rabbits have become an increasingly interesting animal model for the study of lipid metabolism and atherosclerosis [31–35]. Recently, using genome editing techniques, APOE-knockout rabbits have been created as a novel rabbit model for the study of hyperlipidemia and atherosclerosis [36–37]. Compared to wild-type rabbits they showed highly elevated cholesterol and increased triglyceride levels that mimic the symptoms in human cardiovascular disease [36]. Moreover, they showed elevated remnant lipoproteins as well as greater susceptibility to hyperlipidemia with remarkably increased levels of cholesterol and more excessive aortic atherosclerosis [37]. However, the effect of APOE-knockout on rabbits IVD degeneration has not yet been investigated. Therefore, we determined to examine and compare the intensity of IVD degeneration in the lumbar spines of ten APOE-knockout and four wild-type New Zealand White (NZW) rabbits of matching age. Magnetic resonance imaging (MRI) analysis, quantitative analysis of IVD matrix proteins, assays of glucose concentration and viable disc cell numbers were performed, in order to analyse and evaluate the extent of lumbar disc degeneration in each rabbit. Due to the evidence of more advanced IVD degeneration with obstructed lumbar arteries, reduced cell viability and altered matrix protein composition that mimic the symptoms of enhanced IVD degeneration in humans, APOE-knockout rabbits could serve as a valuable animal model for *in vivo* therapeutic approaches of degenerative IVD disorders. Moreover, APOE-knockout rabbits could circumvent the standard surgical interventions that are frequently applied in research animal models for the induction of enhanced IVD degeneration. In addition, they could be used concurrently for therapeutic researches of IVD disorders and atherosclerosis.

## Materials and methods

### Animals and ethical statement

The Central Laboratory Animal Facility of Medical University Innsbruck and all experimental procedures of the study were complied with the Austrian Animal Experimental Act (BGBI. I Nr. 114/2012). The approvals of the Animal Facility (BMWFW-66.011/0017-II/3b/2014) and the experimental procedures were obtained from the National Committee for Animal Care of the Austrian Federal Ministry of Science, Research and Economy. This study is specifically approved by the local research ethics committee (Animal Welfare Body of Medical University of Innsbruck: project 11–2015). APOE-knockout rabbits were recently created [36] and homozygous APOE-knockout mutants were made available to us. Starting with homozygous APOE-knockout mutants, they were bred by the Central Laboratory Animal Facility of Medical University Innsbruck. Ten APOE-knockout rabbits (5 male, 5 female; mean age 2.0 ± 0.3 years; mean weight 4.5 ± 0.6 kg) and four wild-type NZW rabbits (2 male, 2 female; mean age 2.0 ± 0.1 years; mean weight 4.1 ± 0.4 kg) were used for the study. Each rabbit was single housed in a flat deck cage (5400 cm^2^, Scanbur, Denmark) with an elevated platform. Housing conditions were maintained at 18°C, 50% relative humidity and 12/12 h light/dark cycle. Specific Pathogen Free (SPF) quality of the animals was monitored and confirmed according to FELASA recommendations [38]. Commercial standard diet (K-H/V223X, Ssniff^®^, Germany) was fed *ad libitum* and fresh tap water was constantly available. Regular health monitoring and an additional health check proir to anesthesia was performed by the responsible veterinarian.

### MRI analyses of rabbit lumbar spines

Magnetic resonance imaging (MRI) was used to determine the degeneration grades of the lumbar IVDs and measure the rate of contrast agent enhancement within the discs. Ahead of MRI each rabbit was sedated by intramuscular injection of ketamine (35 mg/kg body weight, Ketasol^®^, aniMEDICA GmbH, Germany) and xylazine (5 mg/kg body weight, Xylasol^®,^ aniMEDICA GmbH, Germany). Additionally, a 22-gauge intravenous catheter was placed into a marginal ear vein and a constant infusion of ketamine (130 mg/kg) and xylazine (8 mg/kg) in 0.9% saline of 100 ml mini-bag (B. Braun GmbH, Germany) was administered at a rate of 0.15 ml/min, which provided sufficient anesthesia for up to 2.5 h. There was a constant video monitoring of the anesthetized animal during MRI analysis. MRI of the lumbar spine was performed using a 3T whole-body MRI scanner (Magnetom Verio, Siemens Healthcare). The rabbit was positioned prone on a 24 channel spine array coil and covered with a flexible 8-channel body array coil. All images were acquired in sagittal orientation. T2-weighted (T2w) images were obtained with a fast-spin-echo (FSE) sequence (T2-FSE) (TR/TE = 3000ms/53ms, echo train length: 12, acquisition matrix: 320x236, rectangular FOV: 140mm x 103mm, number of slices: 13, slice thickness: 2mm, interslice gap: 0.2mm, number of averages: 4, voxel size: 0.44mm x 0.44mm x 2mm). For the quantification of T2 relaxation times a multi-echo spin-echo sequence (TR = 1500ms, number of echoes: 16, first TE = 13.5ms, TE increment: 13.5ms, acquisition matrix: 256 x 163, rectangular FOV: 140mm x 111mm, number of slices: 6, slice thickness: 3mm, interslice gap: 3mm, number of averages: 1, voxel size: 0.7mm x 0.5mm x 3mm) was used. For T1-mapping the dual flip angle method [39] based on a fast 3D gradient echo sequence (TR/TE = 5.17ms/2.24ms, flip angle 1 = 2°, flip angle 2:14°, acquisition matrix: 256 x 184, rectangular FOV: 160mm x 160mm, number of slices: 20, slice thickness: 3.6mm, interslice gap: 0mm, number of averages: 2, voxel size: 0.9mm x 0.6mm x 3.6mm) was applied. T2- and T1-maps were calculated automatically by the MRI scanner immediately after image acquisition. For dynamic contrast enhanced imaging of the lumbar spine a high resolution T1-weighted (T1w) 3D gradient echo sequence (3G GE) (TR/TE = 8.8ms/3.0ms, flip angle = 15°, acquisition matrix: 256 x 208, rectangular FOV: 140mm x 114mm, number of slices: 64, slice thickness: 0.5mm, interslice gap: 0mm, number of averages: 3, voxel size: 0.5mm x 0.5mm x 0.5mm) was applied. Prior to injection of the contrast agent Gadobutrol (Gadovist, Bayer Vital, Germany) a set of pre contrast images was obtained with the T1w 3D GE and the T1w FSE sequence. Then Gadobutrol was administered intravenously at a dose of 0.3 mmol/kg body weight and the acquisition of the T1w images was repeated every 10 minutes for at least 120 minutes. After image acquisition the recorded images were transferred to an offline workstation. Image analysis was performed using ImageJ (Wayne Rasband, National Institutes of Health, Bethesda, MD, USA) together with custom written plugins. The Pfirrmann MRI scoring system was used for grading of lumbar disc degeneration on T2w images [40]. For the analysis of the dynamic contrast agent enhancement, regions of interests (ROI) were manually outlined on the midline sections of the lumbar discs (L1/L2-L6/S1). Signal intensities were measured and recorded for every time point by averaging over the respective ROI. Contrast agent enhancements were calculated as changes in signal intensities from baseline divided by the baseline signal intensity. Relative signal changes were plotted as a function of time.

### Recruitment of intervertebral disc tissues

After completing the MRI scan rabbits were deeply anesthetized by increasing the flow rate of the drip to 0.5 ml/min. Afterwards animals were euthanized by intracardiac administration of concentrated potassium chloride and lumbar discs were immediately harvested. Nucleus pulposus (NP) tissue was carefully separated from anulus fibrosus (AF) tissue on the basis of their macroscopic morphology (identification of the innermost lamellar ring of the AF). To avoid contamination of the NP and AF samples great care was taken to exclude surrounding tissues and blood. NP and AF tissues of each rabbit (L1/L2-L6/S1) were separately pooled and finely minced into small fragments of approximately 2 mm^3^. Fractions of NP and AF tissues from each rabbit were used for different experiments.

### Determining the number of viable cells in NP and AF tissues

From each rabbit portions (200 mg) of finely minced NP and AF tissues were separately digested with 0.02% w/v pronase (Sigma-Aldrich) in 5 ml DME/F-12 (Dulbecco’s Modified Eagle’s Medium/Ham’s Nutrient Mixture F12, 1:1 mixture) containing 1% penicillin/streptomycin, 1% glucose and 10% FCS (fetal calf serum) (Sigma-Aldrich) (1 h, 37°C, 5% CO_2_). After filtration of the samples through sterile 75 gm nylon mesh filters (Sigma-Aldrich) and centrifugation of the supernatants (1000 x g, 2 min), pellets were resuspended in 5 ml DME/F-12. NP and AF pellets were digested with 0.02% and 0.04% w/v collagenase II (Sigma-Aldrich) respectively (3 h, 37°C, 5% CO_2_). Samples were filtered through sterile 75 gm nylon mesh filters, supernatants were centrifuged (1000 x g, 2 min) and pellets were resuspended in 1 ml DME/F-12. Using 3-(4,5-dimethylthiazol-2-yl)-2,5-diphenyltetrazolium bromide (MTT) assay the number of viable cells was determined according to the manufacturer’s protocol (MTT Assay Kit, Molecular Probes). Briefly, duplicates of 100 μl samples and duplicates of blanks (100 μl medium alone) were plated into a flat-bottomed 96 well plate and incubated to recover the cells from handling (24 h, 37°C, 5% CO_2_). After adding 10 μl MTT Reagent to each well and incubation for 3 h (37°C, 5% CO_2_), 100 μl of the SDS-HCl solution was added and further incubated for 4 h (37°C, 5% CO_2_). A microtiter plate reader Infinite 200 (TECAN) was used to measure the absorbance in each well at 570 nm. The average value of the blank duplicate readings was subtracted from the average values of the sample duplicate readings and the number of viable cells was calculated from the standard curve. For each sample three independent assays were performed with two replicates.

### Glucose-oxidase test for determination of glucose concentration in NP and AF tissues

For the glucose-oxidase test 200 mg of NP and AF tissues were separately inserted in falcons and rapidly frozen in liquid nitrogen for 1 min. 300 μl of 0.05 M sodium acetate buffer (pH 6.0) containing 4.0 M guanidinium chloride and 0.01 M ethylenediaminetetra-acetate (EDTA) was added to each sample. Samples were shaken for 48 hours at 4°C and centrifuged at 15000 x g (4°C, 30 min). Supernatants were dialyzed against 3 ml sterile deionized water overnight and lyophilized to dryness at 67°C to a constant dry weight. 40 mg of dry weight from each sample was extracted with glucose assay buffer at a concentration of 300 μg/μl. Glucose concentration was determined using glucose-oxidase assay according to the manufacturer’s protocol (Glucose Assay Kit, Abcome). Briefly, a duplicate of 20 μl tissue sample was mixed with 30 μl glucose assay buffer in a flat-bottomed 96 well plate and 50 μl of the glucose reaction mix was added to each well of sample and standard. For serum samples 1 μl of serum was mixed with 49 μl of glucose assay buffer. As background 50 μl of the background control mix (48 μl of assay buffer and 2 μl of glucose probe) was used. Sample, standard and background control were protected from light and incubated for 30 min at 37°C. The absorbance in each well was measured at 570 nm in a microtiter plate reader Infinite 200 (TECAN). The average value of the background duplicate readings was subtracted from the average values of the sample duplicate readings and glucose concentration was calculated from the standard curve. For each sample three independent assays were performed with two replicates.

### Enzyme-linked immunosorbent assay of matrix proteins in NP and AF tissues

For matrix protein isolation NP and AF tissues were treated to constant dry weight as described above. 40 mg of dry weight from each sample was extracted with 300 μl cold radio-immunoprecipitation assay (RIPA) buffer (Sigma-Aldrich) containing inhibitors of protease and phosphatase (Sigma-Aldrich). The mixture was sonicated at 50% pulse for 30 sec, gently shaked on ice for 30 min and centrifuged for 15 min (14000 x g, 4°C). Total protein concentrations were quantified in supernatants using BCA protein assays according to the manufacturer’s protocol (BCA Protein Assay Kit, Life Technologies). The concentrations of matrix proteins in total protein extracts [ng matrix protein / μg total protein] were determined by using enzyme-linked immunosorbent assay (ELISA). The ELISA kits were obtained from R & D Systems (United Kingdom) or Uscn Life Science Inc. (USA) and assays were performed according to the instruction manuals. Aggrecan, collagen II and collagen I as major matrix proteins in NP and AF tissues were analyzed. For each sample three independent assays were performed with two replicates.

### Monolayer expansion and three-dimensional culture of NP and AF cells

NP and AF cells were isolated by successive digestion of NP and AF tissues with pronase and collagenase II as described above. After digestion samples were filtered through sterile 75 gm nylon mesh filters and supernatants were centrifuged (1000 x g, 2 min). Pellets were resuspended in 0.5 ml culture medium DME/F-12 and cultured for 2 weeks in 24 well plates by changing the culture medium every two days (37°C, 5% CO_2_). The monolayer expanded NP and AF cells were harvested by trypsinization and directly used for three-dimensional culture (3D). For the 3D culture a bovine collagen I scaffold of 24 well plate format was used (Viscofan Bioengineering). Briefly, a scaffold was placed into each well that was filled with 250 μl PBS (pH 7.3 without Ca^2+^ / Mg^2+^) and incubated at room temperature for 20 min. The PBS was removed and the plate was left in the operating laminar flow hood overnight. Equilibration of the scaffold was performed for 10 min by adding 250 μl pre-warmed culture medium into each well. NP and AF cells were 3D cultured for two weeks in 500 μl culture medium (37°C, 5% CO_2_) by changing the culture medium every two days. NP and AF cells were then harvested by digestion of the scaffold with 0.04% w/v collagenase II in 250 μl culture medium (37°C, 5% CO_2_, 3 h). Cell suspensions were filtered through sterile 75 gm nylon mesh filter and supernatants were centrifuged (1000 x g, 2 min). Pellets were washed twice with PBS (1000 x g, 2 min) and directly used for expressional analysis of APOE in NP and AF cells.

### Quantitative reverse transcription PCR (RT-qPCR)

To examine the mRNA expression levels of APOE in NP and AF cells of wild type and APOE-knockout rabbits the RT-qPCR was applied. From 3D cultured NP and AF cells total RNA was isolated by using the RNeasy Plus Mini Kit (Qiagen). DNA contamination was removed by DNase 1 (Sigma-Aldrich). The concentration of total RNA was quantified at 260 nm using Biospectrometer (Eppendorf) and equal amounts of RNA were used for reverse transcription (RT). The cDNAs were synthesized using TaqMan Reverse Transcription Reagents (Applied Biosystems) and the mRNA levels of APOE and β-Actine (internal standard) were determined by qPCR using TaqMan gene expression assay (Life Technologies) and LightCycler 480 (Roche Applied Science). The TaqMan Gene Expression Master Mix (1× master mix) supplemented with 200 nM sense, 200 nM antisense primers of APOE, 250 nM APOE-probe and 2 μl of the template DNA was used for PCR reactions in 20 μl of final volume. The PCR program contained an initial denaturation step at 95°C for 15 min, 40 cycles of denaturation at 95°C for 15 s, an extension at 60°C for 1 min, a melt curve stage (65°C to 95°C, increment 0.5°C) and a final extension at 72°C for 10 min. Standard, negative control and sample were run in three replicates of a 96 well plate. The mRNA expression levels were numerically presented using the delta CT (ΔCT) method.

The sequence (5'->3') of the applied primers and probes are listed below:

APOE-sense: AGGAGCTGACCATGCTGATG

APOE-antisense: CTGTTGCACACGTCCTCCAT

APOE-probe: 6FAB-CCATGCTGATGGAGGAGACC-BHQ1

Beta-actin-sense: CAGAAGGACAGCTACGTGGG

Beta-actin-antisense: CATGTCGTCCCAGTTGGTCA

Beta-actin-probe: 6FAB-GACCCTGAAGTACCCCATCG-BHQ1

### Western blot

Western blot was used to examine the protein expression levels of APOE in NP and AF cells of wild type and APOE-knockout rabbits. Total protein was extracted from 3D cultured NP and AF cells by using cold RIPA buffer containing inhibitors of protease and phosphatase. Total protein concentration in supernatants was determined by BCA Protein Assay Kit. Equal amounts of samples protein were separated by sodium dodecyl sulfate polyacrylamide gel electrophoresis (SDS-PAGE, Sigma-Aldrich) and transferred to polyvinylidene fluoride (PVDF) membrane (Merck Millipore). Monoclonal anti-APOE antibody produced in mouse (SAB5300354, Sigma-Aldrich) was used as primary antibody and the interaction between the primary antibody and APOE was detected on the membrane by using horseradish peroxidase-conjugated goat anti-mouse secondary antibody (A3682, Sigma-Aldrich) and Amersham ECL Western Blotting Detection kit (GE Healthcare Life Sciences).

### Statistical data analysis

Landis and Koch based interpretations with κ statistics and agreement percentage among two observers were used for reliable MRI evaluations of lumbar IVD degeneration [40–41]. Statistical data analyses were performed by using the software IBM SPSS Statistics 22 (Armonk New York USA). 1-way ANOVA and pairwise comparisons were used to compare the data of APOE-knockout and wild-type rabbits. Significance was set at p < 0.05.

### Data availability

The authors declare that all data supporting the findings of this study are available within the paper.

## Results

### More advanced grade of degeneration and lower enhancement of Gadobutrol in lumbar IVDs of APOE-knockout rabbits

**Fig 1:**
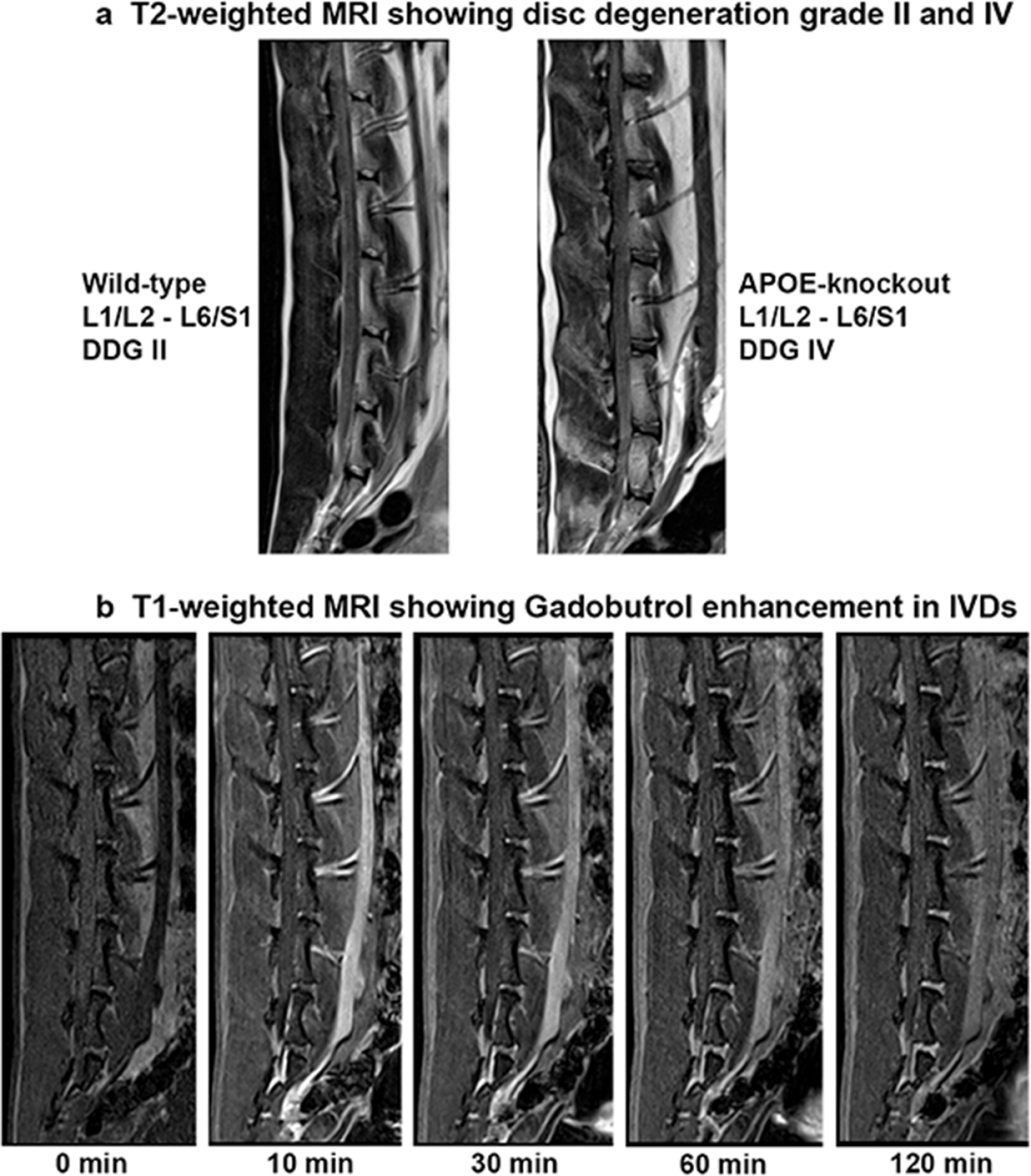
Representative T2- and T1-weighted MRIs showing lumbar IVDs (L1/L2-L6/S1) from two years old wild-type and APOE-knockout rabbits. T2-weighted MRI (Fig 1a) displaying more advanced disc degeneration grade (DDG IV) in the APOE-knockout rabbit as compared to that (DDG II) in the wild-type rabbit. T1-weighted MRI (Fig 1b) illustrating the gradual enhancement of the intravenously administered contrast agent Gadobutrol [0.3 mmol/kg] in lumbar discs observed during a 120-minute period.

**Fig 2:**
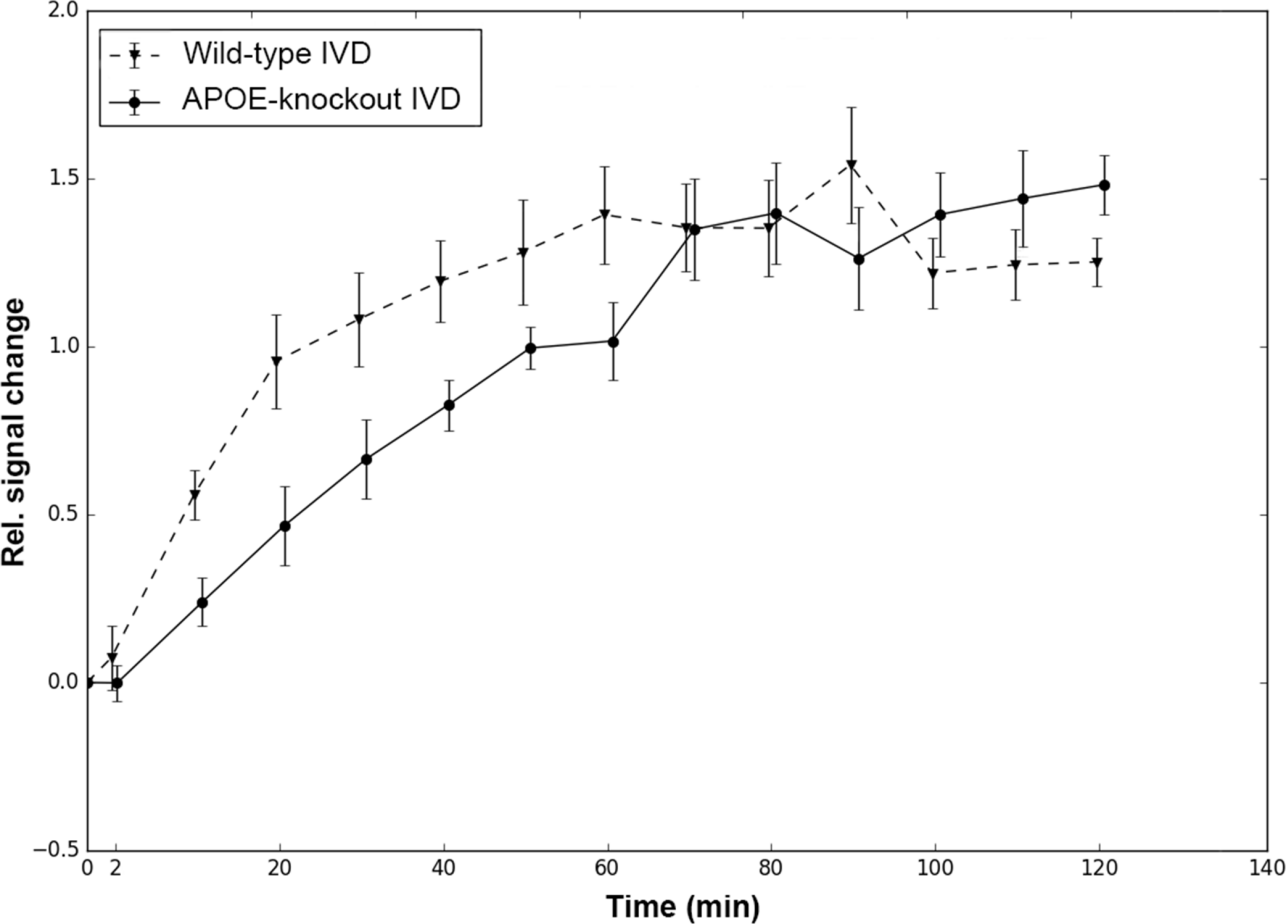
T1-weighted relative signal intensities in IVDs of APOE-knockout and wild-type rabbits. After intravenous administration of Gadobutrol the relative signal intensity was calculated from T1-weighted scans of lumbar discs, as a change in signal intensity from the baseline divided by the baseline signal intensity. The results represent the average values from the lumbar discs (L1/L2—L6/S1) for each given measuring time point (0 to 120 min). For the first 60 min a slower rate of Gadobutrol enhancement was determined in the discs of APOE-knockout rabbits. In wild-type discs clearance of Gadobutrol appeared to start after 60 min, while Gadobutrol enhancement was still going on in APOE-knockout discs.

The T2-weighted MRI signal characteristics of the lumbar IVDs revealed accelerated disc degeneration in APOE-knockout rabbits as compared to that in the wild-type rabbits. The interobserver reliability agreement for the rating of IVD degeneration with two observers was performed as described before [40–41] and the calculated frequency of disagreements were 0.00% with a reliability coefficient κ = 1.00. Consistent score of disc degeneration grade IV (DDG IV) in APOE-knockout rabbits and DDG II in wild-type rabbits were ascertained. Representative images from two years old wild-type and APOE-knockout rabbits show the respective DDG II and DDG IV (Fig 1A). The T1-weighted MRI was used to measure the rate of solute enrichment in the lumbar IVDs. Representative images show a series of T1-weighted scans of lumbar discs that illustrate the gradual enhancement of the low molecular weight Gadobutrol after its intravenous administration (Fig 1B). The relative signal intensity to a given time point (0 to 120 min) was calculated as a change in signal intensity from baseline divided by the baseline signal intensity. The calculated relative signal intensities were plotted as a function of time (Fig 2). Slower rate of Gadobutrol enhancement was verified in lumbar discs of APOE-knockout rabbits and in addition to that no clearance of Gadobutrol was observed during the 120-minute period. The wild-type discs appeared to start with clearance of the contrast agent after 60 minutes, while the APOE-knockout discs were still taking it up.

### Decreased glucose concentration in IVD tissues of APOE-knockout rabbits

**Fig 3:**
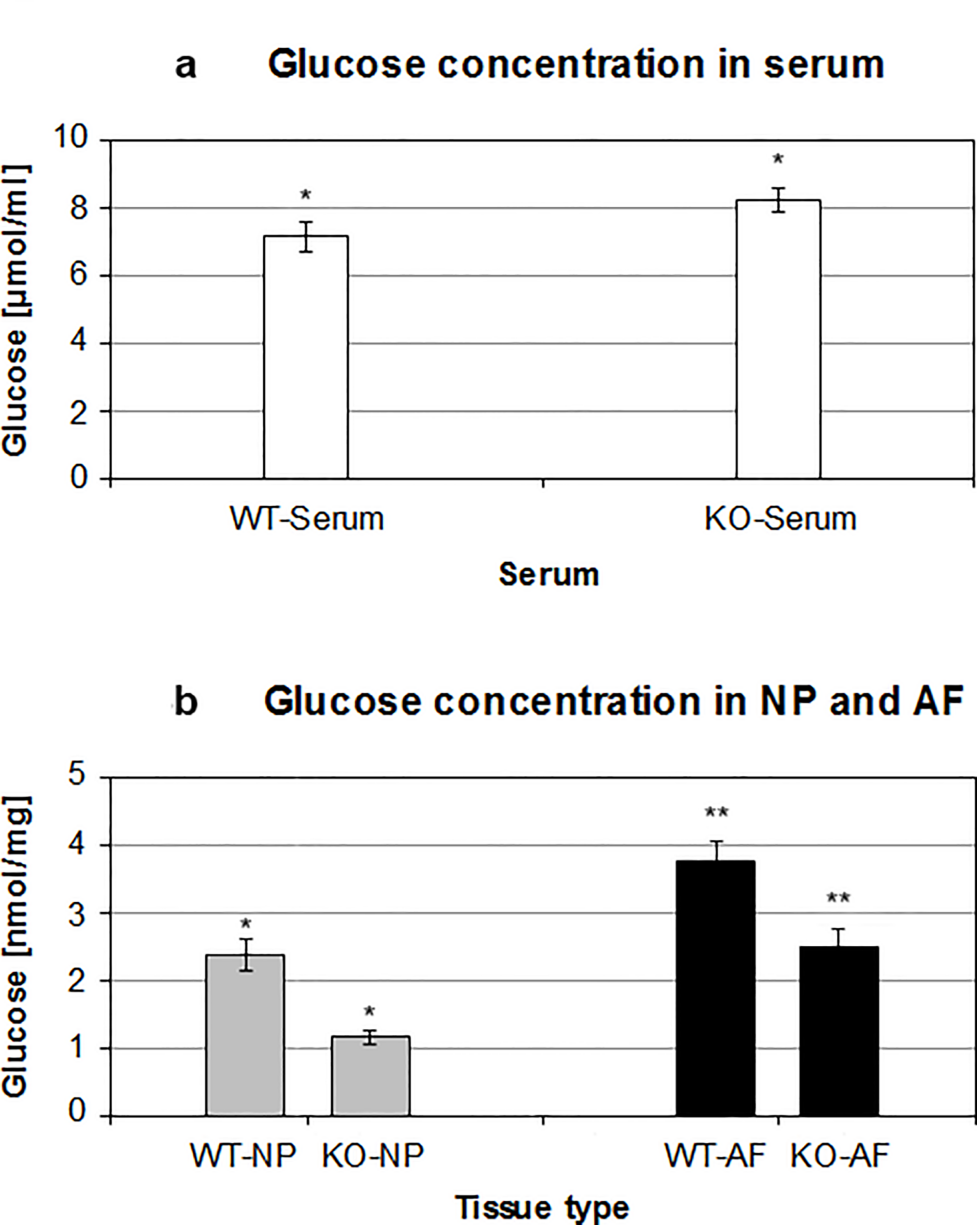
Lower glucose concentration in NP and AF tissues of APOE-knockout rabbits. Using glucose-oxidase assays higher level of glucose concentration in serum samples [μmol/ml] of APOE-knockout rabbits (KO-Serum) was determined as compared to that in serum of wild-type rabbits (WT-Serum) (Fig 3a). On the contrary, the levels of glucose concentration in NP and AF tissues [nmol/mg] of APOE-knockout rabbits (KO-NP, KO-AF) were significantly lower than that in NP and AF tissues of the wild-type rabbits (WT-NP, WT-AF) (Fig 3b). Three independent glucose-oxidase assays were performed in duplicate for each sample and significance was set at p < 0.05.

APOE-knockout induced obstruction of Gadobutrol transport to the lumbar IVDs could also obstruct the nutrient transport, such as glucose. Low concentration of glucose as energy source could badly affect the cellular environment within the discs. Using glucose-oxidase assays the glucose concentration in APOE-knockout and wild-type rabbits were detected. The concentration of glucose was measured in serums [μmol/ml] as well as in NP and AF tissues [nmol/mg] of the rabbits. The recorded mean glucose concentration in serum samples of APOE-knockout rabbits [8.21 ± 0.365 μmol/ml] was 15.3% higher than that of the wild-type rabbits [7.12 ± 0.424 μmol/ml] (p < 0.0001) (Table 1 and Fig 3A). In contrast, the mean glucose concentrations in NP and AF tissues of APOE-knockout rabbits were lower than that of the wild-type rabbits. The glucose concentration in NP of APOE-knockout rabbits [1.14 ± 0.109 nmol/mg] was 48.2% lower than that in NP of wild-type rabbits [2.37 ± 0.240 nmol/mg] (p < 0.0001). Similarly, the glucose concentration in AF of APOE-knockout rabbits [2.48 ± 0.270 nmol/mg] was 33.5% lower than that in AF of wild-type rabbits [3.73 ± 0.319 nmol/mg] (p < 0.0001). Lower glucose concentrations were detected in NP tissues than in AF tissues of the same rabbit group (Table 1 and Fig 3B).

**Table 1:** Glucose concentration levels in serums and IVD tissues of wild-type and APOE-knockout rabbits. Glucose concentrations in serums [μmol/ml] as well as in NP and AF tissues [nmol/mg] were determined using the glucose-oxidase assays. Compared to that in serums of wild-type rabbits (WT-Serum) higher glucose concentration was detected in serums of APOE-knockout rabbits (KO-Serum). In contrast, lower glucose concentrations were confirmed in NP and AF tissues of APOE-knockout rabbits (KO-NP, KO-AF). In addition lower glucose concentrations were detected in NP tissues (WT-NP, KO-NP) than in AF tissues (WT-AF, KO-AF) of the same rabbit group. Three independent glucose-oxidase assays were made in duplicate for each sample and significance was set at p < 0.05.

Tissue	Mean	SD	Mean difference	Change %	P value
WT-Serum	7.12 μmol/ml	0.424	KO—WT = +1.09	+15.3	< 0.0001
KO-Serum	8.21 μmol/ml	0.365			
WT-NP	2.37 nmol/mg	0.240	KO—WT = -1.23	-48.2	< 0.0001
KO-NP	1.14 nmol/mg	0.109			
WT-AF	3.73 nmol/mg	0.319	KO–WT = -1.25	-33.5	< 0.0001
KO-AF	2.48 nmol/mg	0.270			

### Diminished number of viable cells in IVD tissues of APOE-knockout rabbits

**Fig 4:**
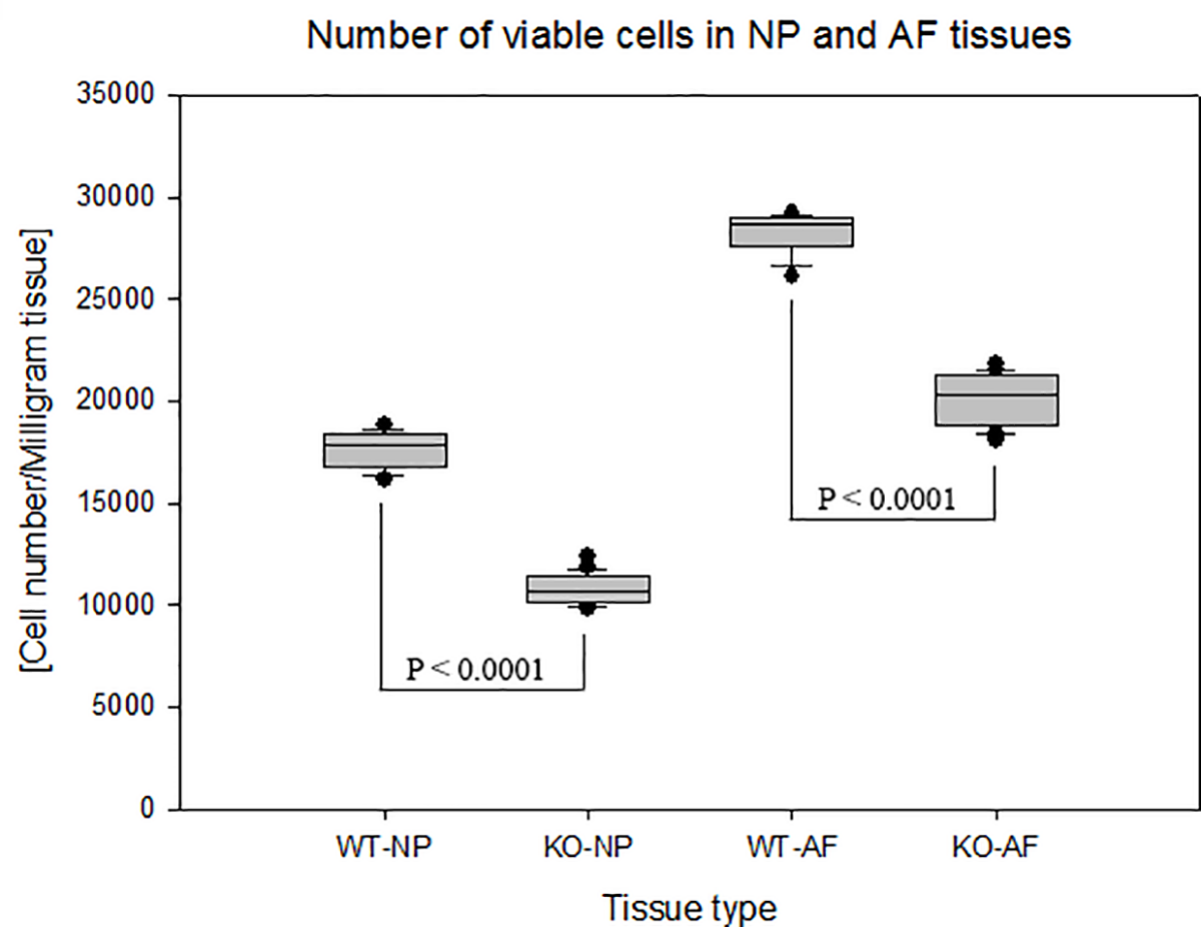
Diminished number of NP and AF cells in APOE-knockout rabbits. MTT assays were used to determine the number of viable cells that were immediately isolated from lumbar NP and AF tissues of APOE-knockout and wild-type rabbits. The recorded number of viable cells per milligram of wet tissue [cell number/milligram tissue] showed lower numbers of viable cells in NP and AF tissues of APOE-knockout rabbits (KO-NP and KO-AF) as compared to that in NP and AF tissues of wild-type rabbits (WT-NP and WT-AF). Box plots with whiskers min. to max. show the number of viable cells per milligram of wet tissue. Three independent MTT assays were performed in duplicate for each sample and significance was set at p < 0.05.

NP and AF cells were directly isolated from lumbar IVDs of wild-type and APOE-knockout rabbits that were immediately harvested after euthanization and their numbers of viable cells were quantified using MTT assays. Decreased numbers of viable cells per milligram of wet tissue [cell number/milligram tissue] were detected in NP and AF tissues of APOE-knockout rabbits. The recorded mean numbers of viable cells in NP and AF tissues of APOE-knockout rabbits were 10867 ± 753 and 20122 ± 1176 respectively; and in wild-type rabbits the respective mean values amounted to 17834 ± 1262 and 28273 ± 1019 (p < 0.0001). Comparing the mean values in wild-type and APOE-knockout rabbits the numbers of viable cells in NP and AF tissues of APOE-knockout rabbits were reduced by 39.1% and 28.8% respectively. In NP tissues of both wild-type and APOE-knockout rabbits lower numbers of viable cells were detected than in the respective AF tissues (Fig 4).

### Altered matrix protein concentration levels in IVD tissues of APOE-knockout rabbits

**Fig 5:**
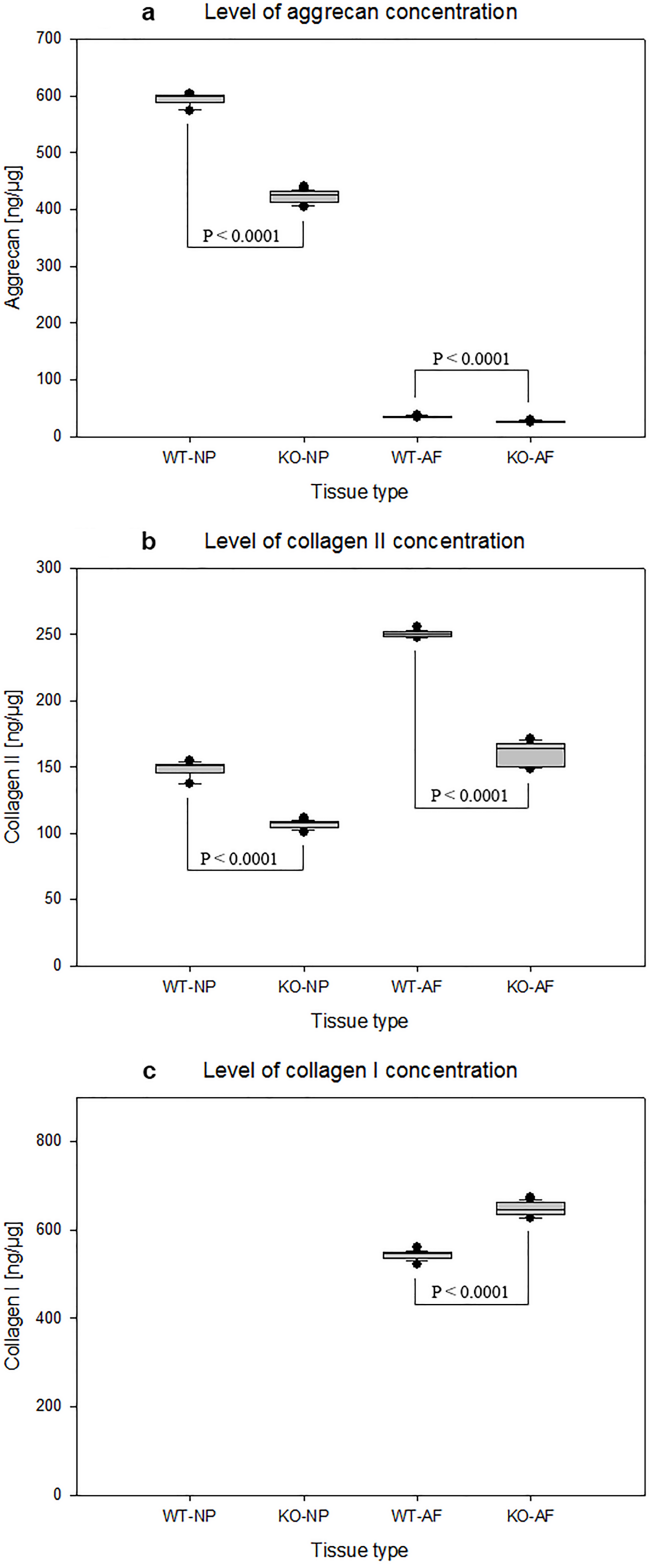
Changed levels of protein concentration in NP and AF tissues of APOE-knockout rabbits. The concentration levels of aggrecan, collagen II and collagen I were determined in total protein [ng/μg] that was extracted from NP and AF tissues of APOE-knockout rabbits (KO-NP and KO-AF) and wild-type rabbits (WT-NP and WT-AF). Decreased concentration levels of aggrecan and collagen II were detected in KO-NP and KO-AF as compared with that in WT-NP and WT-AF; whereas increased level of collagen I was detected in KO-AF as compared with that in WT-AF (p < 0.0001). Collagen I was not detected in both KO-NP and WT-NP. Box plots with whiskers min. to max. show concentrations of aggrecan (Fig 5a), collagen II (Fig 5b) and collagen I (Fig 5c). Three independent ELISA were performed in duplicate for each sample and significance was set at p < 0.05.

The concentration levels of critical extracellular matrix proteins aggrecan, collagen II and collagen I were quantified using ELISA in the total protein extracts of IVD tissues [ng/μg]. In NP and AF tissues of APOE-knockout rabbits decreased levels of aggrecan and collagen II were detected as compared with that in NP and AF tissues of wild-type rabbits. The calculated mean aggrecan concentration levels in NP and AF tissues of APOE-knockout rabbits amounted to 422 ± 10.7 ng/μg and 25.8 ± 1.43 ng/μg respectively. Compared with the respective mean concentration levels (594 ± 11 ng/μg and 35.1 ± 1.4 ng/μg) in NP and AF tissues of wild-type rabbits, the levels of aggrecan in NP and AF tissues of APOE-knockout rabbits were reduced by 28.9% and 26.4% respectively (p < 0.0001) (Table 2, Fig 5A). Similarly, comparing the mean levels of collagen II (106 ± 3 ng/μg and 160 ± 8.07 ng/μg) in NP and AF tissues of APOE-knockout rabbits with the mean levels (148 ± 6.02 ng/μg and 250 ± 2.34 ng/μg) in NP and AF tissues of wild-type rabbits, the levels of collagen II in NP and AF tissues of APOE-knockout rabbits were reduced by 28.3% and 36% respectively (p < 0.0001) (Fig 5B). Although the minimum detectable dose of collagen I in the detection system was very low [0.002 ng/μg], collagen I was not detected in NP tissues of both APOE-knockout and wild-type rabbits. However, high concentrations of collagen I were confirmed in AF tissues of APOE-knockout and wild-type rabbits. Related to the mean concentration level of collagen I in AF tissues of wild-type rabbits (543 ± 9.79 ng/μg), its mean concentration level in AF tissues of APOE-knockout rabbits (647 ± 14.9 ng/μg) was increased by 19.1% (p < 0.0001) (Fig 5C).

**Table 2:** Modified levels of aggrecan, collagen II and collagen I concentrations in NP and AF tissues of APOE-knockout rabbits. NP and AF tissues were isolated from APOE-knockout and wild-type rabbits and total protein was extracted from constant dry weight tissues. The concentration of each matrix protein in total protein extract [ng/μg] was determined using ELISA. Decreased concentration levels of aggrecan and collagen II were confirmed in NP and AF tissues of APOE-knockout rabbits (KO-NP and KO-AF), but increased level of collagen I was determined in KO-AF as compared with the respective values in NP and AF tissues of wild-type rabbits (WT-NP and WT-AF) (p < 0.0001). In both KO-NP and WT-NP collagen I was not detectable. Three independent ELISA were performed in duplicate for each sample and significance was set at p < 0.05.

Matrix Protein	Tissue Type	Mean [ng/μg]	SD	Mean difference [ng/μg]	Change %	P Value
**Aggrecan**	WT-NP	594	11.0	KO–WT = -172	-28.9	< 0.0001
	KO-NP	422	10.7			
	WT-AF	35.1	1.40	KO–WT = -9.30	-26.4	< 0.0001
	KO-AF	25.8	1.43			
**Collagen II**	WT-NP	148	6.02	KO–WT = -42.0	-28.3	< 0.0001
	KO-NP	106	3.00			
	WT-AF	250	2.34	KO–WT = -90.0	-36.0	<0.0001
	KO-AF	160	8.07			
**Collagen I**	WT-NP	-	-	-	-	-
	KO-NP	-	-			
	WT-AF	543	9.79	KO–WT = +104	+19.1	0.0001
	KO-AF	647	14.9			

### Expression of APOE in intervertebral discs

**Fig 6:**
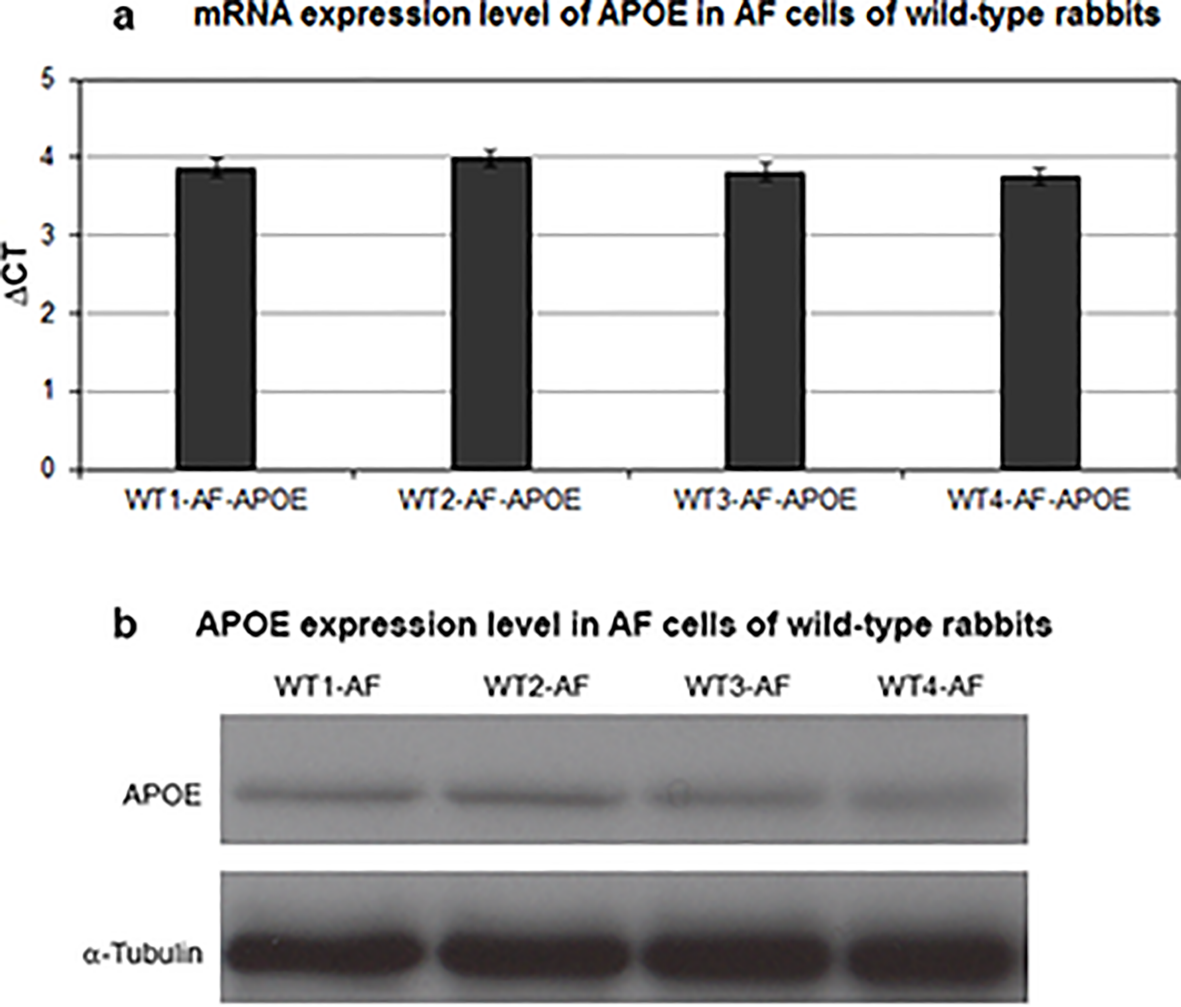
APOE expression in AF cells of wild-type rabbits. Using RT-qPCR and western blot the mRNA and protein expressions of APOE were analyzed in NP and AF cells of wild-type and APOE-knockout rabbits. APOE expression was detected merely in AF cells of wild-type rabbits. Fig 6a and 6b show equivalent APOE mRNA and protein expression levels in AF cells of all tested wild-type rabbits (WT_1-4_-AF). Three independent experiments were performed for each sample and significance was set at p < 0.05.

Previously, the expression of APOE mRNA in several lipoprotein- and non-lipoprotein-producing tissues, such as liver, intestine, spleen, kidney, adrenal gland and brain, were demonstrated [42]. We examined here the expression of APOE in IVD cells of wild-type and APOE-knockout rabbits at mRNA and protein levels by using RT-qPCR and western blot. APOE was detected in the AF cells of wild-type rabbits. The mRNA and protein expression levels were similar in AF cells of all tested wild-type rabbits (Fig 6A and 6B). NP cells of wild-type rabbits did not show any expression of APOE. As expected APOE expression was not detected in any of the APOE-knockout rabbits.

## Discussion

Intervertebral disc degeneration affecting the majority of adults is one of the most common causes of chronic back pain with disability [43, 1–10]. Degenerative changes in IVD emerge as early as the second decade of life, but its exact onset and pace have not been clearly elucidated [44–45]. Alongside other causes of IVD degeneration cardiovascular risk factors and atherosclerosis have received growing attention as one of the plausible causal factors of IVD degeneration. Associations between multiple cardiovascular risk factors and atherosclerosis have been previously described [46–47, 13–16]. Moreover, cardiovascular risk factors such as high levels of cholesterol and triglycerides, which prompt atherosclerosis, have been presumed to be involved in the obstruction of the abdominal aorta and its lumbar branching arteries [25–29]. Nutrient supply to the lumbar vertebrae and their IVDs takes place through the paired lumbar arteries or the middle sacral artery that originate in the abdominal aorta or aortic bifurcation [29]. Formations of excessive fatty streaks and fibrous plaques in the bifurcation and around the orifices of the lumbar branching arteries have been previously shown [25]. Similar to the process of IVD degeneration, atherosclerosis begins to appear early in adult age and its speedy escalation in the abdominal aorta occurs between 44 and 64 years of age [5, 25, 30]. Atherosclerosis mediated barricade of nutrient supply to IVDs could change the cellular and metabolic environment within the discs. This could promote accelerated IVD degeneration that could be accompanied with premature chronic low back pain. It has been recently shown that APOE-knockout rabbits, mimicking the symptoms of atherosclerosis in humans, have great susceptibility to type III hyperlipoproteinemia with remarkably elevated levels of cholesterol, triglyceride, remnant lipoproteins and massive aortic atherosclerosis [36–37]. Since the impact of APOE-knockout on rabbits IVD degeneration has not yet been investigated, we determined to examine and compare disc degeneration in APOE-knockout and wild-type rabbits of matching age.

T2-weighted MRI analysis of the lumbar spines in APOE-knockout rabbits exhibited more advanced disc degeneration grade (DDG IV) as compared with the disc degeneration grade (DDG II) of the wild-type rabbits (Fig 1A). After intravenous injection of the low molecular weight contrast agent Gadobutrol, a series of T1-weighted MRI scans revealed slower enhancement and clearance of Gadobutrol in the IVDs of APOE-knockout rabbits (Fig 2). The slowed enhancement of Gadobutrol could indicate an adversely affected nutrient supply to the IVDs of APOE-knockout rabbits and this obstruction could change the cellular environment within the discs of the APOE-knockout rabbits.

Obstructing the supply of glucose as an energy source could critically affect cell survival and anabolism of extracellular matrix proteins within the discs. Analysis of glucose concentration in IVD tissues of APOE-knockout and wild-type rabbits revealed lower glucose concentration in NP and AF tissues of APOE-knockout rabbits, although higher glucose concentration was detected in their serum samples. Their mean glucose concentrations were reduced by 48.2% in NP tissues and by 33.5% in AF tissues. Comparing the glucose concentrations of AF tissues with NP tissues in the same rabbit group, averagely 54.0% lower glucose concentration was verified in NP tissues of APOE-knockout rabbits and 36.4% lower glucose concentration in NP tissues of wild-type rabbits (Table 1, Fig 3). The pronounced reduction of glucose concentration in NP tissues could be due to the fact that the glucose supply to NP tissues might be additionally repressed by calcification of the endplate, since nutrient supply to the avascular NP occurs only by diffusion through the endplate [48–50].

The number of viable cells and the concentration of extracellular matrix proteins were analysed in IVD tissues of APOE-knockout and wild-type rabbits, as the levels of glucose concentrations could be critical for survival and anabolic activities of IVD cells. Diminished numbers of viable cells were detected in NP and AF tissues of APOE-knockout rabbits. It was reduced by 39.1% in NP tissues and by 28.8% in AF tissues. In NP tissues of both wild-type and APOE-knockout rabbits reduced numbers of viable cells were detected compared to that in AF tissues of the respective rabbits (Fig 4). The reduced number of viable NP cells could be resulted from the repressed supply of glucose through the endplate, which is the central route of solute exchange between the avascular NP tissue and the surrounding blood vessels [48–50]. In a previous study, at least two times less diffusion has been presented in the endplate route than in the perianular route [51]. Our *in vivo* results, which show a tight correlation between the levels of glucose and the number of viable cells, support the results of the previous *in vitro* study, which exhibited the critical effect of glucose deprivation on the maintenance of IVD cell viability [52]. However, an *in vivo* reduction in glucose concentration could occur combined with reductions in oxygen concentration and pH-value, which could apparently worsen the cellular physiology within the discs.

As anabolism of extracellular matrix proteins within IVDs could be affected by the reduction of glucose concentration and disc cell survival, the concentration levels of the main matrix proteins were analyzed in NP and AF tissues of APOE-knockout and wild-type rabbits. Decreased concentration levels of the core matrix proteins aggrecan and collagen II were detected in NP and AF tissues of APOE-knockout rabbits. The mean levels of aggrecan were reduced by 28.9% in NP tissues and by 26.4% in AF tissues. Similarly, the mean levels of collagen II were reduced by 28.3% and 36% respectively. Conversely, the mean level of collagen I was increased by 19.1% in AF tissues of APOE-knockout rabbits (Table 2, Fig 5). These altered concentration levels of the major matrix proteins in NP and AF tissues of APOE-knockout rabbits exhibit the characteristic features of structural and functional deterioration of IVDs, which underline the fundamental problem of disc degeneration.

## Conclusions

In conclusion, lumbar IVDs of APOE-knockout rabbits exhibited accelerated degeneration, lower enhancement of Gadobutrol, decreased concentration of glucose, diminished number of viable cells along with altered concentration levels the major matrix proteins aggrecan, collagen II and collagen I. IVD degeneration induced by APOE-knockout in rabbits could be characterized by structural deterioration that mimics the symptoms of advanced grade disc degeneration in humans. Thus, APOE-knockout rabbits could be used as valuable model for *in vivo* therapeutic approaches in degenerative IVD disorders. Moreover, they can bypass the standard surgical interventions, which are frequently applied in research animal for the induction of enhanced disc degeneration. Also, their parallel use in therapeutic approaches of IVD disorders together with atherosclerosis could contribute to the three principles of Replacement, Reduction and Refinement (3Rs) in animal research [53–54].
